# An *in vitro* model of intestinal infection reveals a developmentally regulated transcriptome of *Toxoplasma sporozoites* and a NF-κB-like signature in infected host cells

**DOI:** 10.1371/journal.pone.0173018

**Published:** 2017-03-31

**Authors:** Pascale S. Guiton, Janelle M. Sagawa, Heather M. Fritz, John C. Boothroyd

**Affiliations:** 1 Department of Microbiology and Immunology, Stanford University School of Medicine, Stanford, California, United States of America; 2 Department of Veterinary Microbiology and Pathology, Washington State University, Pullman, Washington, United States of America; University of Georgia, UNITED STATES

## Abstract

Toxoplasmosis is a zoonotic infection affecting approximately 30% of the world’s human population. After sexual reproduction in the definitive feline host, *Toxoplasma* oocysts, each containing 8 sporozoites, are shed into the environment where they can go on to infect humans and other warm-blooded intermediate hosts. Here, we use an *in vitro* model to assess host transcriptomic changes that occur in the earliest stages of such infections. We show that infection of rat intestinal epithelial cells with mature sporozoites primarily results in higher expression of genes associated with Tumor Necrosis Factor alpha (TNFα) signaling via NF-κB. Furthermore, we find that, consistent with their biology, these mature, invaded sporozoites display a transcriptome intermediate between the previously reported day 10 oocysts and that of their tachyzoite counterparts. Thus, this study uncovers novel host and pathogen factors that may be critical for the establishment of a successful intracellular niche following sporozoite-initiated infection.

## Introduction

*Toxoplasma gondii* is one of the most successful eukaryotic pathogens of medical and veterinary importance, as it can infect humans and a very large number of warm-blooded animals worldwide [1]. Approximately one third of the world’s human population is believed to have been infected with this coccidian, with seroprevalence ranging from 9% to over 80% in different countries [2]. *T*. *gondii* usually causes a mild and self-limiting disease in healthy individuals; however, severe disease sometimes occurs, especially in immunocompromised individuals [3].

There are three developmental forms in the *T*. *gondii* complex life cycle that are key to infection in an intermediate host: sporozoites within sporulated oocysts that are ingested from the environment, rapidly growing tachyzoites that disseminate the infection within a host, and the slowly dividing bradyzoites in tissue cysts that produce the chronic infection [4]. Although tissue cysts can initiate a new infection in a naïve host, epidemiological reports and risk-factor assessments indicate that oocysts are a major source of transmission and are a major public health concern given their prevalence and persistence as environmental contaminants [5–8].

*Toxoplasma* sporozoites have a unique, yet poorly understood biology among *Toxoplasma* developmental stages. Following primary infection with tissue cysts, a single felid host can shed up to 500 million immature oocysts in its feces [9]. Numerous environmental cues initiate maturation and sporulation of shed oocysts, culminating in the production of two sporocysts, that each contains 4 sporozoites encased within a highly impermeable wall [10]. Sporulated oocysts can withstand harsh conditions and persist for extended periods in the environment [6,7,11]. Following ingestion, gastric enzymes and bile degrade the oocyst wall and infective sporozoites are released within the small intestine of an intermediate host where they rapidly invade enterocytes. Once inside the enterocyte, the non-replicating sporozoites convert to tachyzoites that swiftly replicate and disseminate to other organs and tissues [12].

Despite their critical role in transmission and initiation of new *T*. *gondii* infection, the technical challenges associated with the study of oocysts and sporozoites, from their production to handling them in a laboratory setting, have hindered our understanding of the molecular interactions of this developmental form with its host. Notwithstanding these difficulties, comparative transcriptomic and proteomic analyses of *T*. *gondii* oocysts, examined 0, 4 and 10 days after shedding from the cat, have been performed and the results compared to similar data for tachyzoites and bradyzoites [13,14]. These studies showed that while all three infective forms of *T*. *gondii* express genes and proteins known to mediate invasion and pathogenic processes, such as the micronemal protein AMA1 and the rhoptry proteins RON2 and ROP16 [15–19], day 10 sporozoites differentially regulate ~1850 of the ~8000 predicted *Toxoplasma* genes compared to tachyzoites [13], and 20% of their proteome is composed of proteins that are not detected in tachyzoites [14], corroborating the existence of oocyst/sporozoite-specific antigens [20,21]. Radke *et al*. [22] showed that expression of one of these sporozoite-specific surface antigens in tachyzoites, namely sporoSAG, enhances their invasive properties into bovine pulmonary artery endothelial cells. Additionally, Poukchanski *et al*. [23] demonstrated that the sporozoite-specific paralogues of AMA1 and RON2 (“sporoAMA1” and “sporoRON2”, respectively) contribute to host cell invasion during sporozoite infection. Thus, by describing a sporozoite-specific transcriptome and proteome, these studies reaffirmed that although short-lived, sporozoites are biochemically and functionally distinct from tachyzoites and bradyzoites. These prior studies, however, did not examine the impact of sporozoites on the host cells they infect and they looked at the transcriptomes of sporozoites when they have only just completed development and before they have invaded a host cell.

In this report, we used an *in vitro* model of infection of the intestine to profile for the first time the host and parasite transcriptomes during infection with *Toxoplasma* sporozoites. Our studies indicate that sporozoites trigger a NF-κB-like response in rat intestinal epithelial cells (IECs) that mirrors, albeit to a lesser extent, that observed with infection with tachyzoites. We also show that these intracellular sporozoites are in an intermediate transcriptional state between freshly matured sporozoites (day 10) and the tachyzoite form. Together, these findings broaden our understanding of the very first interactions of *Toxoplasma* sporozoites with its host and reveal genes that may mediate fundamental processes of this initial encounter.

## Materials and methods

### Ethics statement

All kitten and mouse experiments were conducted conforming to the guidelines of the American Association for Accreditation of Laboratory Animal Care (AAALAC) protocol and the institutional guidelines set by the Office of Campus Veterinarian at Washington State University (Animal Welfare Assurance A04592). Washington State University AAALAC and institutional guidelines are in compliance with the U.S. Public Health Service Policy on Humane Care and Use of Laboratory Animals. Mice and kittens were maintained at Washington State University (Pullman, WA, USA) in an AAALAC-accredited animal facility. The Washington State University Institutional Animal Care and Use Committee reviewed and approved the animal protocols associated with the current studies. Efforts were made to minimize the numbers of animals used to generate *Toxoplasma* organisms. The kittens used in the study remained healthy throughout. After two weeks of confirmed absence of shedding of *Toxoplasma* oocysts, the kittens were vaccinated and neutered, then adopted out to pre-screened and approved permanent homes.

### Cell culture

Rat non-transformed epithelial cell line IEC-18 [24,25], purchased from the American Tissue Culture Collection (ATCC), was cultured in complete Dulbecco’s modified Eagle’s medium (DMEM; Invitrogen, Carlsbad, CA) supplemented with 10% fetal bovine serum (Hyclone, Logan, UT), 4 mM L-glutamine, 0.1 U/ml bovine insulin, 100 U/ml penicillin, and 100 μg/ml streptomycin, herein referred to as IEC medium. Culture medium was changed twice a week and cells were sub-cultured 1:3 up to the 20^th^ passage according to depositor’s recommendations. African green monkey kidney epithelial cell line MA-104 (ATCC) was maintained in complete DMEM with 10% fetal calf serum, 100 U/ml penicillin, and 100 μg/ml streptomycin.

### Sporozoite excystation

Feces from ~5-month-old kittens infected with *Toxoplasma gondii* type II M4 strain [13] were collected at 5 to 10 days post infection. Approximately 2x10^8^ oocysts were harvested as previously described [13], sporulated for 7 days at room temperature, and stored in 2% sulfuric acid at 4°C for approximately 5 months. On the day of the experiment, 10^8^ sporulated oocysts were washed three times in 1X PBS to remove sulfuric acid. After the final wash, the oocyst pellet was resuspended in 10% Clorox^®^ bleach/PBS and incubated on ice for 30 min. Bleached oocysts were then thoroughly washed twice with 1X PBS and a third time in DMEM media (without serum). The oocyst pellet was then resuspended in DMEM and transferred to a 1.5 ml screw-top microcentrifuge tube containing 350 mg acid-washed glass beads (200–400 mm, Invitrogen) and vortexed at maximum speed in nine 30-second intervals. Approximately 60% of the oocysts were broken open with free sporocysts as determined by visualization under a light microscope. Broken oocysts/sporocysts were collected, spun down, and the pellet was resuspended in DMEM containing 5% sodium taurodeoxycholate hydrate (Sigma, St. Louis, MO). The samples were incubated at 37°C for 10 min to allow sporozoite excystation. Excysted sporozoites were then washed twice in cold DMEM. A third wash was done in DMEM supplemented with 2% FBS. Freshly excysted sporozoites were then resuspended in IEC medium and split into two batches: one (SPZ) with active sporozoites ready for IEC infection and one (fzSPZ) where the sporozoites were inactivated by exposure to 3 cycles of freezing in liquid nitrogen (-196°C) for 3 min and thawing at 37°C in a water bath for 3 min.

Tachyzoites were also derived from M4 sporozoites from the same oocyst harvest described above. Freshly extracted sporozoites were used to infect confluent monolayers of MA-104 cells in complete DMEM at 37°C with 5% CO2. Following egress, tachyzoites were passaged and maintained in culture in MA-104 cells until used to infect IECs.

### *In vitro* model of infection of intestinal epithelium

**Fig 1:**
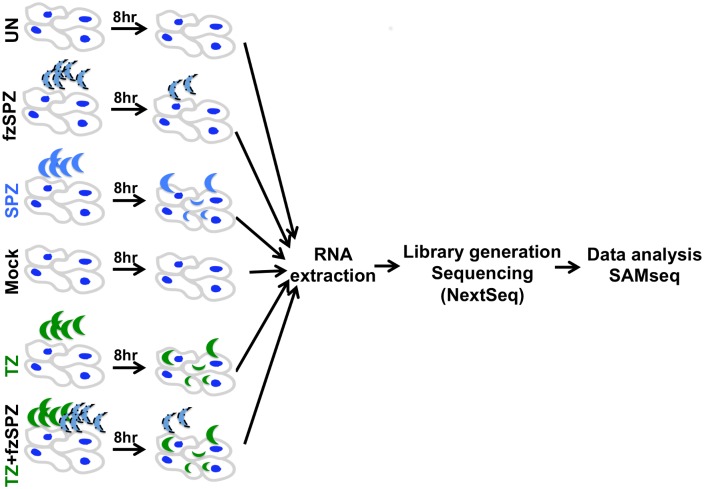
Experimental layout for *in vitro* infection of the intestine. Confluent rat intestinal epithelial cells (IEC-18) were infected with either sporozoites (SPZ) or tachyzoites in the absence (TZ) or presence (TZ+fzSPZ) of frozen-thawed sporozoites for 8 hours at 37°C. As a control for possible effects of oocyst/sporocyst wall components and MA-104 cells debris, sporozoites inactivated by freezing (fzSPZ) or MA-104 cell lysates (Mock) were added to IEC-18 cells, respectively. All experiments were performed in biological duplicate (i.e., starting with individual populations of sporozoites) and with two technical replicates. Total RNA was extracted and RNA sequencing was performed using the Illumina NextSeq platform. SAMseq analysis was used to identify differentially regulated genes of both host and parasite origin in various pairwise comparisons.

Two independent experiments were performed with two technical replicates per experiment. All infections were performed at 37°C for 8 hours in IEC-18 cells. The specific conditions for our study, depicted in Fig 1, are described below.

1. *Uninfected IEC-18 (UN)*: 25,000 IEC-18 cells were seeded in each well of 12-well tissue culture-treated plates in IEC medium and incubated for 48 hours to reach confluence (approximately one million cells).
2. *Frozen-thawed sporozoites control (fzSPZ)*: Two million frozen-thawed sporozoites were added to a well of confluent IEC-18 obtained as described in 1.
3. *Active sporozoites infection (SPZ)*: Two million freshly excysted sporozoites, estimated from hemocytometer counts, were used to infect a well with confluent IEC-18. The target nominal multiplicity of infection (MOI) was 2. Confluent IEC-18 on glass coverslip in a well of 24-well plate was infected in parallel for MOI determination.
4. *Mock infection (Mock)*: Uninfected confluent MA-104 cells were washed in IEC medium, scraped, syringe-lysed through a 27-gauge needle, and passed through a 5-μm filter. MA-104 filtered lysate was added to confluent IEC-18 in a volume matching the tachyzoite inoculum.
5. *Active tachyzoites infection (TZ)*: MA-104 cells infected with M4 tachyzoites for 48 hours were scraped, syringe-lysed through a 27-gauge needle, and passed through a 5-μm filter. Tachyzoites were counted on a hemocytometer, and 1 million were used to infect IEC-18 cells in IEC medium. Parallel infection of confluent IECs on glass coverslips were used for MOI determination.
6. *Active infection with tachyzoites in presence of frozen-thawed sporozoites control (TZ+fzSPZ)*: Tachyzoites, prepared as above, were used to infect IEC-18 in presence of frozen-thawed sporozoite material (prepared as described above). This condition controls for any effect the oocyst/sporocyst wall debris might have on the host cell and/or tachyzoite transcriptomic profiles.

Note that infections with tachyzoites were done subsequently to the sporozoite infections after determination of the number of sporozoites excysted from oocyst harvest.

### Infection quantification

The effective MOI for active sporozoites and tachyzoites was assessed at 8 hours post infection (hpi) using a Red/Green invasion assay [26] modified as follows: At 8 hpi, infected monolayers on glass coverslips were fixed with 4% formaldehyde (Polysciences Inc, Warrington, PA) in 1X PBS for 10min at room temperature. Samples were then washed three times in 1X PBS for 5 min and stored at 4°C in 1X PBS until staining. Samples were blocked in 3% BSA/PBS. Extracellular and attached parasites were stained as follow: blocking buffer was replaced with 3% BSA/PBS containing polyclonal rabbit antisera raised against *T*. *gondii* (1:1500 dilution) and samples were incubated for 1h at 37°C. After washing samples as described above, Alexa Fluor 594 (red) conjugated goat anti-rabbit (Molecular Probes, Eugene, OR) was added at 1:2000 in 3% BSA/PBS for 1 hour at 37°C. To gain access to intracellular parasites, samples were washed three times as above and permeabilized with 0.1% Triton-X100 in 3% BSA/PBS for 30 min at room temperature. Samples were washed once with 1X PBS for 5 min and then stained with polyclonal mouse antibody against *T*. *gondii* in 3% BSA/PBS at 1:1500 for 1 hour at 37°C. Samples were washed thrice and stained with FITC (green) conjugated goat anti-mouse antibody (Molecular probes) at 1:2000 in 3% BSA/PBS for 1 hour at 37°C. After two PBS washes at room temperature, 5000X DAPI nuclear stain was added to 1X PBS (1:2000) and used to stain samples for 1 min at room temperature. Samples were washed, mounted, and visualized at 40x with the EVOS^®^ FL Auto cell imaging system (Invitrogen). Extracellular parasites (red), intracellular parasites (green), and host cells (DAPI-stained nuclei) were enumerated from 25 random fields/coverslip using the EVOS^®^ FL Auto software and the values obtained were used to determine the average MOI for each infection at 8 hpi (effective MOI).

### RNA extraction, library preparation, and sequencing

At 8 hpi, 1 ml TRIzol reagent (Invitrogen) was added to each well. Lysates were collected into RNAse/DNAse-free Eppendorf tubes and frozen at −80°C. RNA extraction for all 24 samples was performed on the same day. Total RNA was extracted following the manufacturer’s instructions, with some modifications. Frozen samples were thawed on ice and equilibrated at room temperature. 0.2 ml chloroform was added to TRIzol suspensions, which were then mixed for 15 seconds. Tubes were incubated for 3 min at room temperature and then spun at 12,000 rpm for 15 min at 4°C. RNA in the aqueous phase was transferred into a fresh tube and 0.5 ml absolute isopropyl alcohol was added. Each tube was inverted three times and incubated at room temperature for 10 min. They were then spun at 12,000 rpm for 20 min at 4°C. After decanting the supernatants, RNA pellets were washed with 1 ml 75% ethanol. Tubes were inverted to mix by hand and then spun at 12,000 rpm for 20 min at 4°C. Supernatants were removed and the RNA pellets were air-dried in open tubes for approximately 10 min. The RNA pellets were resuspended in 25 μl RNase-free DEPC-water (with concentrations ranging from ~180 to ~470 ng/μl). RNA samples were submitted to the Stanford University Functional Genomic Facility (SFGF) for purity analysis using the Agilent 2100 Bioanalyzer. Multiplex sequencing libraries were generated with RNA Sample Prep Kit (Illumina) according to manufacturer’s instructions and pooled for a single high-throughput sequencing run using the Illumina NextSeq platform (Illumina Nextseq 500 model instrument). Illumina NextSeq sequencing generated on average ~24 million reads for each sample (Table A in S1 File).

### Mapping and differential expression analysis

Raw reads were uploaded onto the CLC Genomics Workbench 8.0 (Qiagen) platform for independent alignments against the genomes of *Rattus norvegicus* (Ensembl.org/Rnor.6.0) and *Toxoplasma* Type II Me49 strain (ToxoDB-24, Me49 genome). All parameters were left at their default values. The number of reads that mapped to the *R*. *norvegicus* and *T*. *gondii* genomic reference files are listed in Table A in S1 File and each gene in each reference genome and the corresponding number of reads mapped in each sample are listed in Tables B and C in S1 File for the host and parasite, respectively.

Many genes are so highly conserved across evolution that they have sequences that are almost identical between *Toxoplasma* and rat. This makes it difficult to know exactly which reads in a given sample from infected cells derive from the *Toxoplasma* vs. rat versions of the gene. Because of this, we needed to identify and exclude such genes from our analysis. To do this, we first searched the uninfected and mock-infected RNASeq data for reads mapping to the *Toxoplasma* genome; because these samples were uninfected, any such reads would indicate spurious matches. We then compared the number of such reads to the number for the same gene in the infected samples where *Toxoplasma* infection is present. After normalizing total read numbers to be the same for each sample, any *Toxoplasma* gene that in the uninfected controls had ≥20% of the number of reads in the TZ or SPZ samples was deemed compromised and so it was excluded from all downstream analyses. *Toxoplasma* genes that had an average number of reads in the uninfected samples <20% of the average adjusted reads in the infected sample were left in the analysis but the read numbers from the infected sample were adjusted by subtracting the average number of reads in the uninfected and mock samples, after normalization for total read number. Genes with less than 5 exon reads mapping to the rat genome or to the parasite genome in all samples were excluded from further analysis. The number of total reads mapped to each genome after the adjustments described above was used to determine the RPKM (Reads Per Kilobase of transcript per Million mapped reads), rounded to the nearest one-tenth value, as the relative expression for each rat and *Toxoplasma* gene in each sample (Tables D and E in S1 File). SAMseq [27] package for the R platform was used to identify genes with significant changes between two samples. To identify genes with statistically different expressions between samples, we set the delta (Δ) value at 10% FDR (False Discovery Rate) with *q*-value less than 5%. All the *q*-values obtained from SAMseq analyses are listed in Table F in S1 File. Only genes with *q*-value less than 5% and an average of at least 5 exon reads in one of the two conditions being compared were considered for further analysis. Among these genes, only those with RPKM ratios ≥1.5 for the two samples being compared and consistent in the two infections with tachyzoites (“TZ” and “TZ+fzSPZ”) were included in the list of differentially expressed genes. Lastly, we manually curated the lists of differentially regulated host genes obtained at the end of this analysis pipeline to exclude host genes where the read number might be substantially influenced by *Toxoplasma* reads. This was done by first creating a merged file of the rat and *Toxoplasma* transcribed genomes. The reads from the tachyzoite-infected sample were then mapped to this “merged” genome (the program searches for the parasite or host gene with the best match to each read) and to the rat genome alone. Any gene where the number of reads mapping to the rat genome in the merged set dropped by ≥10% relative to the number that mapped to the rat genome alone, indicating significant presence of *Toxoplasma* mRNA corresponding to this conserved gene, was excluded from further analysis. In practice, this resulted in excluding host genes encoding tubulin, actin, ABCB4, and Rack1-201 as genes where we could not eliminate the possibility that *Toxoplasma* mRNA was substantially contributing to the read number.

### Gene Set Enrichment Analysis (GSEA)

Gene Ontology (GO) for rat genes was obtained from the Rat Genome Database (available at http://rgd.mcw.edu) and Rat Ensembl (http://uswest.ensembl.org).

GSEA [28,29], which is available through the Broad Institute at http://www.broadinstitute.org/gsea/index.jsp, was the pathway analysis software we used to determine whether defined sets of differentially expressed rat genes in our experiment show statistically significant overlap with gene sets in the curated Molecular Signatures Databases (MsigDB) Hallmark gene set collection and an enrichment for a specific pathway [30].

Gene identification and gene product descriptions for *Toxoplasma* were obtained from ToxoDB release 29 (ToxoDB.org) and from published reports [31–33]. Metabolic Pathway Enrichment tool available on ToxoDB release 29 was used to determine enrichment in *Toxoplasma* differentially expressed gene sets.

## Results

### *In vitro* infection of intestinal epithelial cells and parameters for RNAseq analysis

Following ingestion by an intermediate host, sporozoites are excysted and rapidly invade intestinal epithelial cells (IECs) [12]. To study the initial stages of oocyst-initiated *Toxoplasma* infection, we employed an *in vitro* model using the IEC-18 cell line from rats and the experimental design depicted on Fig 1. As a source of parasites, we used sporulated oocysts from feces of kittens experimentally infected with *Toxoplasma* M4 strain (type II) [13]. This is the same strain we previously used to study oocyst development (days 0, 4 and 10 post-shedding in the feces) but in the current study, the oocysts had been sporulated for 7 days, stored for 5 months in 2% sulfuric acid at 4°C and washed in phosphate-buffered saline just prior to use, all standard conditions for storage and recovery of viable oocysts.

**Fig 2:**
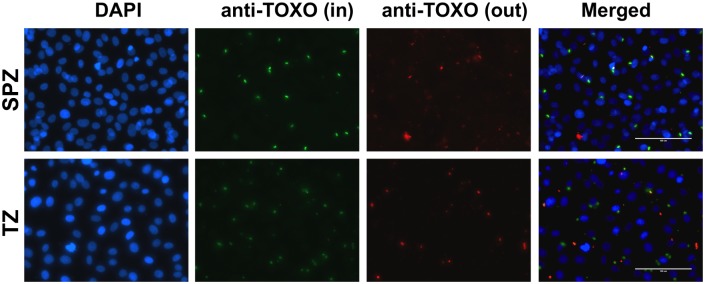
Quantification of infection in IEC-18 cells exposed to sporozoites or tachyzoites. Representative fluorescent microscopy images of confluent IEC-18 cells on glass coverslips infected with *Toxoplasma* type II M4 strain sporozoites and tachyzoites for 8 hours. Parasites were stained with either mouse or rabbit anti-*Toxoplasma* antibody before and after membrane-permeabilization to identify extracellular parasites (red) and intracellular parasites (green). DAPI was used to stain nuclear DNA. Images were obtained at 40X magnification. The scale bar is 100 μm.

To mimic *in vivo* conditions, the washed oocysts were treated with sodium taurodeoxycholate hydrate, an anionic detergent similar to bile salts, to release the sporozoites within and then two million of such excysted sporozoites were used to infect one million non-transformed IEC-18 cells for 8 hours (“SPZ”). The 8-hour infection timeframe was chosen to maximize invasion of the sporozoites but minimize conversion of the sporozoites to tachyzoites and replication once inside the cell; previous workers have shown that sporozoite-to-tachyzoite conversion occurs about 12 hours after infection as assessed by expression of tachyzoite-specific surface markers [34]. As a control to identify host transcriptomic changes that might result simply from exposure to oocyst/sporocyst wall debris or other sporozoite-specific pathogen-associated molecular patterns (PAMPs), IECs were exposed to sporozoites rendered non-infectious by 3 cycles of freezing in liquid nitrogen (-196°C) and thawing at 37°C (“fzSPZ”) which are known to destroy their infectivity [11,35,36]. Since much is known about the effect of infection with tachyzoites and to enable comparisons between infections with the two stages, we also infected the IECs with 1 million syringe-lysed M4 tachyzoites (TZ) in the presence or absence of fzSPZ (“TZ” and “TZ+fzSPZ,” respectively). We used half the number of tachyzoites compared to sporozoites as preliminary experiments revealed that the infectivity of the sporozoites in these conditions is about half that of tachyzoites. Parallel infections on cover-slips and subsequent invasion assays (Fig 2) revealed that the mass infections used for RNA preparation had actual multiplicities of infection for the sporozoites and tachyzoites of 0.18 and 0.26, respectively. While not exactly the same, these were judged sufficiently close to allow comparison between the two datasets. Lastly, and as a further control for nonspecific effects, mock infections of the IECs were performed using syringed lysates from uninfected MA-104 cells (Mock). All infections and controls were performed in quadruplicate.

RNA was extracted at 8 hpi for each of the six conditions depicted in Fig 1 and all 24 samples were submitted for RNA sequencing in a single lane using the NextSeq platform. We independently mapped the sequenced reads to the genomes of *Rattus norvegicus* and the *Toxoplasma* type II Me49 strain (Tables A-C in S1 File) and identified differentially expressed genes for all pairwise comparisons. For this analysis, a gene was considered to be differentially expressed if: 1) it had at least 5 exon reads in at least one of the conditions being compared; 2) its relative expression (Reads Per Kilobase of transcript per Million mapped reads or RPKM) between two samples being compared was statistically significant by SAMSeq [27], a computational method specifically designed for analysis of RNAseq data (using a *q*-value cut-off less than 5% at 10% FDR, Table F in S1 File); 3) its RPKM showed at least 1.5-fold difference between the samples being compared; and 4) when compared to infection with tachyzoites, the difference observed was not affected by the presence of fzSPZ. Details of data processing are outlined in the Materials and Methods.

### *Toxoplasma* sporozoites trigger an NF-kB-like signature response in IEC-18 cells

Given that the oocyst wall includes complex polysaccharides, proteins, and acid-fast lipids [37], the first pairwise analysis we performed was to check for possible pathogen-associated molecular patterns (PAMPs) in the oocyst-derived preparations of sporozoites. To do this, we compared the RNAseq results for uninfected IEC-18 cells vs. those exposed to the fzSPZ. The results showed no significant differences using the criteria described above (Table F in S1 File) and so we conclude that, at least in the conditions being used here, there are no major PAMPs detected by IEC-18 cells in sporozoite preparations derived from oocysts.

To determine the transcriptional changes that occur in IECs in response to sporozoite infection, we next compared the transcriptome of IEC-18 cells infected with sporozoites to fzSPZ-exposed IECs (although we saw no significant differences between the fzSPZ and the uninfected control, it was nevertheless the most appropriate control). Of the 14616 rat genes analyzed (Table D in S1 File), only 26 genes showed ≥1.5-fold difference between the SPZ-infected and fzSPZ controls (Table 1). This indicates that infection with sporozoites does not trigger overwhelming transcriptional changes in IECs, at least at the time point used here, 8 hpi, and recognizing that the MOI was only 0.18 and so 82% of cells in the sample were not infected. The most striking characteristic of this set of 26 genes (Table 1), however, is that gene set enrichment analysis (GSEA from the Broad Institute [28–30]) revealed that at least 21 of the 26 genes are involved in the host inflammatory responses (Table 2). Specifically, GSEA revealed that 19 of these 26 genes are associated with TNF-α signaling via NF-*κ*B (*q*-value = 4.9 x 10^−39^ at 5% FDR), including *Nfκb2*, *Nfκbiα*, and *Nfκbiε*, and 6 that are associated with the interferon gamma response (*q*-value = 1.7x10^-8^ at 5% FDR), namely *Nfκbiα*, *Vcam1*, *Tnfaip2*, *Tnfaip3*, *Ccl7*, and *Ccl2*. *Tnfsf18*, which is associated with the TNF-α signaling, was also up-regulated in SPZ-infected cells but is not included in the curated reference gene sets of the molecular signatures database (MSigDB Hallmark collection from the Broad Institute [30]) as a “hallmark” of inflammatory responses. Similar to *Tnfsf18*, the acid-sensing G-protein coupled pH-sensing receptor *Gpr68* (LOC102553138, also known as *Ogr1*) [38] and the Rab GTPase *Rab32* were among the 26 host genes up-regulated by sporozoite infection and these proteins have previously been shown to be important in host immune responses as, respectively, a regulator of intestinal inflammation via TNF-mediated NF-*κ*B signaling [39], and restriction of intracellular bacterial pathogens [40–43]. Moreover, five of the 26 genes encode inflammatory chemokines, namely *Ccl20*, *Cxcl1*, *Ccl2*, *Ccl7*, and *Csf1*, which showed an increase in expression relative to the fzSPZ controls ranging from approximately 2- (*Ccl7* and *Csf1*) to 18-fold (*Ccl20*). Together, these findings indicate that infection with sporozoites triggers a significant NF-*κ*B-like inflammatory response in intestinal epithelial cells in the initial stages of infection.

**Table 1:** Rat genes with significantly higher expression in IECs upon infection with sporozoites.

		RPKM						FOLD CHANGE			
Gene ID	Description	UN	fzSPZ	SPZ	Mock	TZ	TZ+fzSPZ	Mock/UN	SPZ/fzSPZ	TZ/Mock	TZ+fzSPZ/Mock
**AABR07021465.2**	**Novel LincRNA**	0.2	0.1	2.1	0.2	4.8	4.9	1.0	**21.0**	**24.0**	**24.5**
***Ccl20***	**chemokine (C-C motif) ligand 20**	0.1	0.1	1.8	0.1	1.2	1.3	1.0	**18.0**	**12.0**	**13.0**
***Vcam1***	**vascular cell adhesion molecule 1**	0.2	0.1	1	0.1	3	2.5	0.5	**10.0**	**30.0**	**25.0**
***Gpr68***	**LOC102553138, G protein-coupled receptor 68**	0.3	0.1	0.7	0.2	1	1.1	0.7	**7.0**	**5.0**	**5.5**
***Cxcl1***	**chemokine (C-X-C motif) ligand 1**	3.4	3.2	14.9	2.8	15.9	17.2	0.8	**4.7**	**5.7**	**6.1**
***Ccl2***	**chemokine (C-C motif) ligand 2**	19	15	61.4	22.3	163	168.5	1.2	**4.1**	**7.3**	**7.6**
***Egr1***	**early growth response 1**	8.1	6.2	21.2	5.2	19.2	18.6	0.6	**3.4**	**3.7**	**3.6**
***Egr2***	**early growth response 2**	1	0.7	2.3	1.9	4.6	5	1.9	**3.3**	**2.4**	**2.6**
***Relb***	**v-rel oncogene homolog B**	2.8	2.5	7.8	2.9	10.7	10.6	1.0	**3.1**	**3.7**	**3.7**
***Nfkbia***	**Nuclear factor kappa B inhibitor alpha**	8.3	7.6	22.6	7.6	26.6	26.2	0.9	**3.0**	**3.5**	**3.4**
***Tnfaip3***	**tumor necrosis factor alpha-induced protein 3**	2.6	2.3	5.6	3	10.5	10.3	1.2	**2.4**	**3.5**	**3.4**
***Lif***	**leukemia inhibitory factor**	7.3	6	14	8.8	27.7	28.1	1.2	**2.3**	**3.1**	**3.2**
***Tnfsf18***	**tumor necrosis factor superfamily member 18**	1.9	1.9	4.4	1.7	4.6	5	0.9	**2.3**	**2.7**	**2.9**
***Nfkbie***	**Nuclear factor kappa B inhibitor epsilon**	4.1	3.3	7.6	4.5	12	12.2	1.1	**2.3**	**2.7**	**2.7**
***Olah***	**oleoyl-ACP hydrolase**	2.1	1.9	4	2.3	5	5.2	1.1	**2.1**	**2.2**	**2.3**
***Btg2***	**BTG family member 2**	2.5	2	4.2	2.9	5.5	5.8	1.2	**2.1**	**1.9**	**2.0**
***Nfkb2***	**nuclear factor of kappa B subunit 2**	9.4	8.6	17.4	9.6	22.9	23	1.0	**2.0**	**2.4**	**2.4**
***Ccl7***	**chemokine (C-C motif) ligand 7**	6.8	5.5	11.1	8.9	23.1	24.8	1.3	**2.0**	**2.6**	**2.8**
***Junb***	**jun B proto-oncogene**	26	20.7	37	26.8	51.6	50.5	1.0	**1.8**	**1.9**	**1.9**
***Dusp5***	**dual specificity phosphatase 5**	4.1	3.5	6.1	5.8	9.1	9.6	1.4	**1.7**	**1.6**	**1.7**
***Olr1***	**oxidized low density lipoprotein receptor 1**	20.9	18.1	31.3	21.9	47.4	46	1.0	**1.7**	**2.2**	**2.1**
***Tnfaip2***	**tumor necrosis factor alpha-induced protein 2**	25.1	21.5	35.2	32.1	48.4	48.8	1.3	**1.6**	**1.5**	**1.5**
***Csf1***	**colony stimulating factor 1 (macrophage)**	17.7	15.7	25.4	21.7	41.2	42.3	1.2	**1.6**	**1.9**	**1.9**
***Rcan1***	**regulator of calcineurin 1**	5.7	4.9	7.8	5.3	7.1	7.6	0.9	**1.6**	1.3	1.4
***Rnf19b***	**ring finger protein 19B**	6.6	6.1	9.4	7.1	11.6	11.5	1.1	**1.5**	**1.6**	**1.6**
***Rab32***	**member RAS oncogene family**	11.2	10.2	14.9	12.3	21.4	22.4	1.1	**1.5**	**1.7**	**1.8**

Rat genes with significantly higher (≥1.5 fold) expression in SPZ vs. fzSPZ (ranked from highest to lowest fold-change in SPZ-infected IECs relative to the fzSPZ control). The values for all conditions are shown as well as fold change for experimental vs. control samples.

**Bold** = *q*-value ≤5%; non-bold = *q*-value >5% and fold change <1.5 over controls.

**Table 2:** Host pathways significantly enriched in IECs infected with sporozoites or tachyzoites.

	SPZ infection		TZ infection	
Pathways (# genes in reference set)	# genes	*q*-value	# genes	*q*-value
**TNFa signaling via NF-kB (200)**	**19**	**4.91x10**^**-39**^	**38**	**3.89x10**^**-63**^
**Inflammatory response (200)**	**8**	**2.84x10**^**-12**^	**19**	**9.12x10**^**-25**^
**Interferon gamma response (200)**	**6**	**1.74x10**^**-8**^	**16**	**1.04x10**^**-19**^
**Allograft rejection (200)**	**4**	**4.1x10**^**-5**^	**9**	**3.29x10**^**-9**^
Complement (200)	4	4.1x10^-5^	-	-
**p53 pathway (200)**	**3**	**8.7x10**^**-4**^	**10**	**1.63x10**^**-10**^
Epithelial mesenchymal transition (200)	3	8.7x10^-4^	-	-
KRAS signaling up (200)	3	8.7x10^-4^	-	-
**UV response up (158)**	**3**	**6.2x10**^**-4**^	**13**	**2.96x10**^**-16**^
**IL6 JAK STAT3 signaling (87)**	**3**	**1.22x10**^**-4**^	**9**	**2.8x10-**^**12**^
Interferon alpha response (97)	-	-	7	9.84x10^-9^
Apoptosis (161)	-	-	9	1.15x10^-8^
IL2 STAT5 signaling (200)	-	-	9	3.29x10^-9^

Only the top 10 pathways based on increasing *q*-value at 5% FDR are shown.

Ranked from lowest to highest *q*-value in SPZ infection.

**Bold =** pathways enriched in both infections.

### The response of IEC-18 cells infected with tachyzoites parallels that of sporozoite-infected cells

To determine how the host response to sporozoite infection differs from infection with tachyzoites, we wanted to compare our SPZ data to IEC-18 cells infected with M4 tachyzoites (“TZ”). As the control for these infections we used IEC-18 cells exposed to scraped, syringed lysates from uninfected cells (i.e., “mock-infected”) since lysed mammalian cells can be a source of danger-associated molecular patterns (DAMPs, like ATP). The results showed 105 host genes with significantly higher expression in IECs infected with tachyzoites relative to the mock infection, using the same criteria as described above for the SPZ analysis: 25 of these genes are the same as the genes that were higher in the SPZ-infected host cells as listed in Table 1; additional genes with the greatest up-regulation in the TZ-infected IECs not listed in Table 1 are shown in Table 3 while the remaining 52 of the 105 affected genes are in Table G in S1 File. GSEA showed that over 50% of these 105 up-regulated genes are involved in host immune responses (Table 2), including 38 up-regulated genes associated with TNF-α signaling via NF-κB (*q*-value = 3.9x10^-63^ at 5% FDR) and 16 with IFNγ-induced signaling (*q*-value = 1x10^-19^). These results are in agreement with previous reports [44–46] showing that tachyzoites induce NF-κB activation and downstream signaling during infection.

**Table 3:** Rat genes with significantly higher expression in IECs infected with tachyzoites but not with sporozoites.

		RPKM						FOLD CHANGE			
Gene ID	Description	UN	fzSPZ	SPZ	Mock	TZ	TZ+fzSPZ	Mock/UN	SPZ /fzSPZ	TZ /Mock	TZ+fzSPZ /Mock
***Csf2***	**colony stimulating factor 2**	0	0	0.6	0	1.1	1.2	N/A	*inf*	**inf**	**inf**
**AABR07005779.5**	**novel lincRNA**	0	0	0.1	0	0.3	0.4	N/A	*inf*	**inf**	**inf**
***Cd69***	**CD69 molecule**	0	0	0.3	0.1	2.1	2	inf	*inf*	**21.0**	**20.0**
***Cx3cl1***	**chemokine (C-X3-C motif) ligand 1**	0.2	0.2	1.9	0.2	3.3	2.6	1.0	*9*.*5*	**16.5**	**13.0**
***Traf1***	**TNF receptor-associated factor 1**	0.1	0.1	0.6	0.1	0.7	0.7	1.0	*6*.*0*	**7.0**	**7.0**
***Snrpg***	**small nuclear ribonucleoprotein polypeptide G**	1.6	1.4	1.7	0.3	1.5	2.2	0.2	1.2	**5.0**	**7.3**
***Gbp7***	**LOC685067, guanylate binding protein family member 6**	0.1	0.1	0.1	0.1	0.5	0.4	1.0	1.0	**5.0**	**4.0**
**RGD1311892**	**similar to hypothetical protein FLJ10901**	0	0	0.1	0.1	0.4	0.3	inf	*inf*	**4.0**	**3.0**
***Birc3***	**baculoviral IAP repeat containing 3**	0.5	0.6	1.4	0.5	1.9	1.7	1.0	*2*.*3*	**3.8**	**3.4**
***Fos***	**FBJ murine osteosarcoma viral oncogene homolog**	0.5	0.4	0.9	0.4	1.3	1.1	0.8	*2*.*3*	**3.3**	**2.8**
***Cxcl10***	**chemokine (C-X-C motif) ligand 10**	4.4	3.6	7.3	6.2	18.8	17.8	1.4	*2*.*0*	**3.0**	**2.9**
***Bdkrb1***	**bradykinin receptor B1**	0.8	0.8	1.7	0.7	2.1	2.4	0.9	*2*.*1*	**3.0**	**3.4**
***Cxcl6***	**chemokine (C-X-C motif) ligand 6**	0.3	0.2	0.4	0.2	0.6	0.6	0.7	*2*.*0*	**3.0**	**3.0**
***Nox1***	**NADPH oxidase 1**	2.3	1.9	3.3	3.4	8.2	8	1.5	*1*.*7*	**2.4**	**2.4**
***Tnfsf9***	**tumor necrosis factor superfamily member 9**	1.1	1.3	1.4	1	2.2	1.9	0.9	1.1	**2.2**	**1.9**
***Atf3***	**activating transcription factor 3**	0.6	0.7	1.1	0.6	1.3	1.1	1.0	*1*.*6*	**2.2**	**1.8**
***Zmynd15***	**zinc finger MY0-type containing 15**	1.3	1	1.7	1.5	3.2	3	1.2	*1*.*7*	**2.1**	**2.0**
***Prr5l***	**proline rich 5 like**	0.9	0.8	1.3	0.8	1.7	1.9	0.9	*1*.*6*	**2.1**	**2.4**
***Tgif2***	**TGFβ-induced factor homeobox 2**	3.1	3.1	5.9	2.4	5	4.7	0.8	*1*.*9*	**2.1**	**2.0**
***Gem***	**GTP binding protein overexpressed in skeletal muscle**	3.5	3	5	2.6	5.4	5.6	0.7	*1*.*7*	**2.1**	**2.2**
***Icam1***	**intercellular adhesion molecule 1**	22.5	20.4	30.6	19.7	39.7	41	0.9	*1*.*5*	**2.0**	**2.1**
***Cish***	**cytokine inducible SH2-containing protein**	1.3	1.3	2.1	0.9	1.8	1.7	0.7	*1*.*6*	**2.0**	**1.9**
***Rhbdf2***	**rhomboid 5 homolog 2**	2.8	2.4	3.1	2.2	4.4	4.3	0.8	1.3	**2.0**	**2.0**

Only rat genes that are significantly higher in TZ vs. Mock with ≥ 2-fold increase are shown.

Ranked from highest to lowest fold-change in TZ-infected IECs relative to the Mock control.

**Bold** = *q*-value ≤5%; *italicized* = *q*-value >5% but fold change ≥1.5; non-bold = *q*-value >5% and fold change <1.5 over controls; inf = infinity; N/A = not applicable since dividing zero by zero.

Even though we saw no evidence of PAMPs in the frozen sporozoite material (“fzSPZ”), we also asked if the host response with TZ would be affected by the presence of such material. To do this, we compared the results with TZ alone to the results obtained when the IECs were infected with TZ in the presence of fzSPZ (“TZ+fzSPZ”). This comparison revealed no host genes whose expression was significantly different between the two samples (Table F in S1 File), confirming that the fzSPZ contains no major PAMPs that significantly affect the transcriptome of IECs, at least in the 8 hours of infection used here.

The above results enabled us to next compare the host transcriptomic response from TZ-infection with that seen for SPZ-infection. To simplify the analysis, we restrict our discussion to comparing TZ- and SPZ-infected cells. Specifically, we compared the 105 host genes whose expression was altered by TZ-infection relative to the mock-infected control with the results of SPZ-infection relative to the fzSPZ control. The results showed that 25 of the 26 genes that were higher in the SPZ-infected samples relative to the fzSPZ controls described above are also among the 105 genes significantly altered during TZ-infection (Table 1). Note that *Rcan1*, which was only 1.3-fold up in the TZ-infection and therefore did not meet our threshold for inclusion of being at least 1.5-fold up, was just 1.6-fold up in the SPZ-infected samples (Table 1); this marginal difference is unlikely to be biologically significant. Additionally, 38 of the 80 remaining genes with increased expression in cells infected with tachyzoites relative to Mock control also showed higher expression in the SPZ infection relative to the fzSPZ control, but not to a statistically significant degree. This set of 38 includes *Cd69*, *Cx3cl1*, and *Traf1*, which are listed in Table 3. Together, these findings indicate that, similar to their response during SPZ-infection, TZ-infection of IECs elicits a NF-κB-like inflammatory response. At least in these experiments however, both in terms of the number of genes affected and the magnitude of the effect, infection with tachyzoites appeared to elicit a stronger response (Tables 1–3).

We did not observe significant reduction in expression of any rat genes in IEC-18 cells infected with either sporozoites or tachyzoites.

### Intracellular sporozoites are transcriptionally different from maturing sporozoites in day 10 sporulated oocysts

In addition to data on differences in the host transcriptome, which was the primary objective of this study, our dataset allowed us to assess transcriptomic differences between tachyzoites and the sporozoites at the time point used here, 8 hpi. Previous analyses have compared tachyzoites and sporozoites but the sporozoites used in those prior experiments were extracellular and from oocysts that were in the process of maturation (i.e., at most just 10 days after being shed from the kittens). We were interested, therefore, in analyzing the transcriptomes of the intracellular sporozoites used here that have been given 5 months to complete their development, albeit at 4°C, and have been intracellular for up to 8 hours.

The first observation was that, compared to the day 10 (D10) sporozoites, these intracellular sporozoites have massively down-regulated expression of genes involved in oocyst/sporocyst formation, such as the oocyst wall protein, TgOWP2, and the hypothetical tyrosine-rich wall proteins, as well as a gene encoding the late-embryogenesis domain containing protein, TgERP (Table 4) [13,14,47,48]. This is as expected since the need for expression of these genes will have long passed (oocyst formation appears complete by 10 days after shedding in that the sporozoites inside are fully formed and infectious). Unexpectedly, however, we made similar observations for several genes involved in sporozoite attachment and invasion, including the surface antigen sporoSAG (Table 4), and the putative moving junction components, sporoAMA1 and sporoRON2, which were readily detected in D10 oocysts [13,14]. Specifically, we observed an average RPKM of 27 for sporoSAG in the SPZ-infected material compared to previous RNAseq data (ToxoDB) that showed this gene to be abundantly expressed in D10 oocysts with RPKM values of approximately 2293 (Table 4). We did not detect any transcript for sporoAMA1 and sporoRON2 in the intracellular sporozoites (Table C in S1 File) whereas they had RPKM values of approximately 217 and 31, respectively, in D10 sporulated oocysts (Table 4). These observations indicate that the sporozoites in the present study have distinct transcriptomic profiles from sporozoites given just 10 days to sporulate.

**Table 4:** Expression dynamics of oocyst-associated genes from unsporulated oocysts to intracellular sporozoites.

		Oocysts/extracellular sporozoites			Intracellular parasites		
Gene ID	Description	D0	D4	D10	SPZ	TZ	TZ+fzSPZ
TGME49_281590	hypothetical protein (15.5% Tyr)	8.4	84249.9	9246.8	0	16.5	18.4
TGME49_237080	hypothetical protein (6.2% Tyr)	25464.7	3539.0	6295.3	0	0	0
TGME49_227100	hypothetical protein	146.9	8598.7	4482.7	92.3	21.1	23.5
TGME49_319890	hypothetical protein (5.5% Tyr)	0.0	26799.0	4020.6	0	0	0
TGME49_202100	hypothetical protein	19301.6	2803.8	4013.2	0	0	0
TGME49_202110	hypothetical protein	23302.1	1862.6	3243.5	0	0	0
TGME49_259900	hypothetical protein, conserved	1.1	11901.5	3087.5	0	3	8.2
TGME49_320530	hypothetical protein (5.6% Tyr)	3.7	4647.5	2493.1	9.2	11.9	15.4
TGME49_258550	SRS28 (SporoSAG)	3.8	9879.1	2292.7	27	7.7	17.2
TGME49_276850	LEA (TgERP)	2.4	8201.0	2215.5	0	0	0
TGME49_320540	hypothetical protein	3428.4	2282.0	2026.3	0	0	0
TGME49_294600	hypothetical protein	12.0	2631.5	1831.2	0	0	0
TGME49_204520	hypothetical protein	134.6	1531.8	925.4	8.3	14.2	13.8
TGME49_276880	hypothetical protein (LEA)	10.0	3105.7	752.6	0	0	0
TGME49_316190	superoxide dismutase, putative (SOD3)	1.1	1481.8	630.2	0	0	0
TGME49_229320	haloacid dehalogenase-like hydrolase domain-containing protein	2022.7	223.0	514.6	0	0	0
TGME49_270950	hypothetical protein	10.4	533.9	433.4	0	0	0
TGME49_209610	oocyst wall protein OWP2	3538.6	310.0	411.6	0	14.7	16.4
TGME49_287250	hypothetical protein (13.5% Tyr)	1999.5	243.7	367.7	4.3	6.4	8.2
TGME49_266860	BTB/POZ domain-containing protein	821.3	195.9	334.7	0	0	0
TGME49_202090	hypothetical protein	2992.1	168.0	307.1	0	0	0
TGME49_272240	hypothetical protein	1.7	407.8	280.6	0	0	0
TGME49_205090	hypothetical protein	165.5	229.9	222.7	0	0	0
TGME49_315260	alanine dehydrogenase, putative	59.1	126.4	197.6	41.1	3.2	2.7
TGME49_215885	hypothetical protein	1.0	21.5	19.1	0	0	0
							
TGME49_31573^a^	sporoAMA1	114.8	880.5	216.8	0	0	0
TGME49_26512^a^	sporoRON2	0.5	49.1	31.1	0	0	0

Levels of expression (RPKM) in intracellular sporozoites and tachyzoites during IEC infection of the previously reported top 25 oocyst genes with higher expression in D10 sporozoites compared to tachyzoites and bradyzoites from Fritz *et al*. [13].

Ranked from highest to lowest RPKM in D10 sporulated oocysts.

RPKM values for D0, D4, and D10 oocysts obtained from ToxoDB.

^*a*^ Sporozoite-associated genes not part of the top 25 genes described above.

### The transcriptomic profile of infecting sporozoites is distinct from that of tachyzoites

Next, we compared the transcriptomic data on the intracellular sporozoites with the data for the intracellular tachyzoites. The results showed that of the 6469 *Toxoplasma* genes evaluated (Table E in S1 File), there were 743 genes with significantly higher expression in the SPZ vs. TZ samples (Table H in S1 File). Table 5 provides the list of the top 50 such genes based on fold-increase over TZ.

**Table 5:** Top 50 genes with significantly higher expression in sporozoites compared to tachyzoites.

		RPKM			FOLD CHANGE	
Gene ID	Description	SPZ	TZ	TZ+fzSPZ	SPZ/TZ	SPZ/TZ+fzSPZ
TGME49_203682	**hypothetical protein**	62.7	0	0	**inf**	**inf**
TGME49_203688	**hypothetical protein**	662.8	0	0	**inf**	**inf**
TGME49_235010	**hypothetical protein**	17.7	0	0	**inf**	**inf**
TGME49_265538	**hypothetical protein**	68.4	0	0	**inf**	**inf**
TGME49_274140	**hypothetical protein**	204.4	1.6	5.2	**127.8**	**39.3**
TGME49_247500	**acyl-CoA dehydrogenase middle domain-containing protein**	48.7	1.2	0	**40.6**	**inf**
TGME49_239260	**histone H4**	170.1	4.3	4.8	**39.6**	**35.4**
TGME49_203685	**hypothetical protein**	341.1	10.4	11.6	**32.8**	**29.4**
TGME49_253030	**glycosyl hydrolase, family 31 protein**	349.4	12.3	15	**28.4**	**23.3**
TGME49_289027	**hypothetical protein**	74	3	5	**24.7**	**14.8**
TGME49_217530	**hypothetical protein**	486.2	21.7	29.5	**22.4**	**16.5**
TGME49_305160	**histone H2Ba**	54.6	2.6	2.9	**21.0**	**18.8**
TGME49_315480	**acyl-CoA dehydrogenase middle domain-containing protein**	105.9	5.4	6	**19.6**	**17.7**
TGME49_286460	**hypothetical protein**	100.1	6.1	13.6	**16.4**	**7.4**
TGME49_315340	**SAG-related sequence SRS52C**	31	2.2	3.7	**14.1**	**8.4**
TGME49_268985	**hypothetical protein**	94.7	6.8	11.3	**13.9**	**8.4**
TGME49_222940	**hypothetical protein**	23.5	1.7	5.6	**13.8**	**4.2**
TGME49_246995	**hypothetical protein**	31.2	2.3	1.9	**13.6**	**16.4**
TGME49_227050	**ATPase domain-containing protein**	115.7	8.7	7.6	**13.3**	**15.2**
TGME49_286040	**hypothetical protein**	40.3	3.1	3.5	**13.0**	**11.5**
TGME49_315260	**alanine dehydrogenase**	41.1	3.2	2.7	**12.8**	**15.2**
TGME49_310260	**hypothetical protein**	220.3	17.6	12.2	**12.5**	**18.1**
TGME49_231960	**dense granule protein GRA28**	535.1	42.8	46.3	**12.5**	**11.6**
TGME49_314330	**ABC transporter, ATP-binding domain-containing protein**	72.2	5.9	6.6	**12.2**	**10.9**
TGME49_309760	**hypothetical protein**	28.6	2.4	4.1	**11.9**	**7.0**
TGME49_313440	**hypothetical protein**	28.8	2.5	4.1	**11.5**	**7.0**
TGME49_250220	**hypothetical protein**	153.9	15.4	22	**10.0**	**7.0**
TGME49_276860	**hypothetical protein**	35.6	3.8	5.1	**9.4**	**7.0**
TGME49_203720	**vitamin k epoxide reductase family protein**	314.9	33.7	35.7	**9.3**	**8.8**
TGME49_300020	**ATP-dependent metallopeptidase HflB subfamily protein**	145.2	17.2	17.3	**8.4**	**8.4**
TGME49_244260	**hypothetical protein**	129	16.2	14.5	**8.0**	**8.9**
TGME49_240310	***Toxoplasma gondii* family E protein**	46.2	6	5.7	**7.7**	**8.1**
TGME49_279350	**hypothetical protein**	158.7	20.7	19.7	**7.7**	**8.1**
TGME49_244408	**hypothetical protein**	46.3	6.6	3.7	**7.0**	**12.5**
TGME49_210310	**hypothetical protein**	59.3	8.7	7.5	**6.8**	**7.9**
TGME49_201850	**WD domain, G-beta repeat-containing protein**	43.9	6.7	12	**6.6**	**3.7**
TGME49_227660	**DNA methyltransferase 2, putative**	29.6	4.6	3.8	**6.4**	**7.8**
TGME49_243720	**peroxisomal biogenesis factor PEX11**	25	4	3	**6.3**	**8.3**
TGME49_227610	**hypothetical protein**	95.9	15.9	15.6	**6.0**	**6.1**
TGME49_315910	**hypothetical protein**	115.3	19.5	15	**5.9**	**7.7**
TGME49_320270	**hypothetical protein**	12.3	2.1	2.4	**5.9**	**5.1**
TGME49_238073	**hypothetical protein**	59.7	10.2	17.1	**5.9**	**3.5**
TGME49_252350	**hypothetical protein**	10.5	1.8	4	**5.8**	**2.6**
TGME49_203230	**hypothetical protein**	23.2	4	2.2	**5.8**	**10.5**
TGME49_237860	**protein kinase domain-containing protein**	47.1	8.2	8.5	**5.7**	**5.5**
TGME49_301240	**hypothetical protein**	25.3	4.5	8	**5.6**	**3.2**
TGME49_323000	**KRUF family protein**	69.1	12.5	13.9	**5.5**	**5.0**
TGME49_202790	**dihydrouridine synthase (dus) protein**	25.8	4.7	8.8	**5.5**	**2.9**
TGME49_288685	**Fe-S protein assembly co-chaperone HscB protein**	70.4	12.9	12.5	**5.5**	**5.6**
TGME49_270760	**asparagine synthase**	32.1	6.1	5.9	**5.3**	**5.4**

Top 50 based on fold change SPZ/TZ.

Ranked from highest to lowest fold-change in SPZ/TZ.

**Bold** = *q*-value<5% at 10% FDR; inf = infinity.

On the other hand, 1485 genes were lower in SPZ compared to TZ using the criteria described above; however, given that there were only ~43% as many total *Toxoplasma* reads in the SPZ relative to the TZ samples and to increase the confidence with which we called genes that are significantly higher in TZ vs. SPZ, only those genes with at least 20 reads in TZ among the 1485 are listed in Table I in S1 File. Table 6 lists the top 50 genes of these 999 genes based on fold-change. From this comparative analysis, we have identified three functionally related sets of genes that differ between the intracellular sporozoites from tachyzoites: genes encoding secreted proteins, those involved in gene expression and cell division, and those related to metabolism. These will be presented individually, below.

**Table 6:** Top 50 genes with significantly higher expression in tachyzoites compared to sporozoites.

		RPKM			FOLD CHANGE	
Gene ID	Description	SPZ	TZ	TZ+fzSPZ	TZ/SPZ	TZ+fzSPZ/SPZ
**TGME49_271930**	**hypothetical protein**	0	250.4	214.1	**inf**	**inf**
**TGME49_323310**	**hypothetical protein**	0	154.3	123.3	**inf**	**inf**
**TGME49_235690**	**hypothetical protein**	0	128.1	100.3	**inf**	**inf**
**TGME49_255450**	**hypothetical protein**	0	109.3	96.5	**inf**	**inf**
**TGME49_230480**	**hypothetical protein**	0	108.3	117.3	**inf**	**inf**
**TGME49_280570**	**SAG-related sequence SRS35A**	0	108.1	94.5	**inf**	**inf**
**TGME49_286590**	**SPM2**	0	97	70.8	**inf**	**inf**
**TGME49_218270**	**hypothetical protein**	0	93.1	89.2	**inf**	**inf**
**TGME49_322110**	**hypothetical protein**	0	86.2	110.7	**inf**	**inf**
**TGME49_238150**	**hypothetical protein**	0	80.2	79.4	**inf**	**inf**
**TGME49_315750**	**hypothetical protein**	0	60.7	49.4	**inf**	**inf**
**TGME49_206690**	**GAPM2B**	0	56.6	60.5	**inf**	**inf**
**TGME49_267680**	**MIC12**	0	50	42	**inf**	**inf**
**TGME49_313780**	**hypothetical protein**	0	47.3	46.1	**inf**	**inf**
**TGME49_202390**	**S15 sporozoite-expressed protein**	0	44.1	39.3	**inf**	**inf**
**TGME49_245670**	**PDHE1A**	0	43.6	40.3	**inf**	**inf**
**TGME49_234380**	**hypothetical protein**	0	42.7	44.2	**inf**	**inf**
**TGME49_242570**	**hypothetical protein**	0	42.4	54	**inf**	**inf**
**TGME49_286580**	**hypothetical protein**	0	41.7	39.8	**inf**	**inf**
**TGME49_225690**	**hypothetical protein**	0	41.3	40	**inf**	**inf**
**TGME49_224530**	**IMC5 (ALV11)**	0	39.6	38.6	**inf**	**inf**
**TGME49_268680**	**hypothetical protein**	0	39.4	31.9	**inf**	**inf**
**TGME49_282200**	**ATPase, AAA family protein**	0	37.8	34.7	**inf**	**inf**
**TGME49_229280**	**hypothetical protein**	0	37	41.2	**inf**	**inf**
**TGME49_209170**	**hypothetical protein**	0	36.3	38.8	**inf**	**inf**
**TGME49_221990**	**hypothetical protein**	0	36.3	32.9	**inf**	**inf**
**TGME49_232780**	**hypothetical protein**	0	35.9	34	**inf**	**inf**
**TGME49_239830**	**TBC domain-containing protein**	0	35	31.1	**inf**	**inf**
**TGME49_278780**	**hypothetical protein**	0	33.3	31.8	**inf**	**inf**
**TGME49_242100**	**hypothetical protein**	0	32.1	35.7	**inf**	**inf**
**TGME49_294400**	**hypothetical protein**	0	29.7	26.8	**inf**	**inf**
**TGME49_224000**	**hypothetical protein**	0	29.6	25.9	**inf**	**inf**
**TGME49_218910**	**hypothetical protein**	0	29.5	30.1	**inf**	**inf**
**TGME49_244500**	**Tubulin-tyrosine ligase family protein**	0	29.5	31.1	**inf**	**inf**
**TGME49_221250**	**hypothetical protein**	0	28.9	22.7	**inf**	**inf**
**TGME49_264660**	**SAG-related sequence SRS44**	0	28.7	30.9	**inf**	**inf**
**TGME49_225020**	**hypothetical protein**	0	25.7	19.1	**inf**	**inf**
**TGME49_247250**	**RbAp46**	0	24	24.1	**inf**	**inf**
**TGME49_309410**	**AP2XI-1**	0	22	25.9	**inf**	**inf**
**TGME49_292375**	**KRUF family protein**	0	21.5	22.8	**inf**	**inf**
**TGME49_233770**	**calcium-translocating P-type ATPase**	0	20	16.2	**inf**	**inf**
**TGME49_218362**	**zinc finger protein ZFP1**	0	19.5	19.7	**inf**	**inf**
**TGME49_217700**	**AP2XII-2**	0	19.4	16.8	**inf**	**inf**
**TGME49_201250**	**histone lysine methyltransferase, SET, putative**	0	18.5	23.3	**inf**	**inf**
**TGME49_208020**	**AP2Ib-1**	0	16.7	15	**inf**	**inf**
**TGME49_201230**	**kinesin motor domain-containing protein**	0	16.2	12	**inf**	**inf**
**TGME49_217860**	**hypothetical protein**	0	14	14	**inf**	**inf**
**TGME49_276920**	**protein phosphatase 2C domain-containing protein**	0	13.9	14.8	**inf**	**inf**
**TGME49_203830**	**FHA domain-containing protein**	0	13	9.3	**inf**	**inf**
**TGME49_223060**	**MORN repeat-containing protein**	0	13	14	**inf**	**inf**

Top 50 based on fold change TZ/SPZ.

Ranked from highest to lowest RPKM in TZ.

**Bold** = *q*-value <5% at 10% FDR; inf = infinity.

### Shared and distinct sets of genes encoding secreted proteins

*Toxoplasma* proteins derived from the specialized secretory organelles, namely micronemes, rhoptries, and dense granules, are critical for invasion, intracellular growth and modulation of host responses. These organelles are all present in sporozoites, albeit in somewhat different numbers relative to their abundance in tachyzoites [49]. The 4241 transcripts that showed no significant difference between sporozoites and tachyzoites during infection of IECs (Table E in S1 File) included genes encoding well-characterized secreted proteins (Table 7), which are known to facilitate parasite invasion (RON2 and RON4), contribute to the formation of the parasitophorous vacuole (GRA2), or modulate host responses to *Toxoplasma* tachyzoites (ROP5, ROP16, ROP18) [50–53]. This finding suggests that the recently invaded sporozoites and tachyzoites examined here share a subset of key proteins that may be critical for the intracellular lifestyle of *Toxoplasma*.

**Table 7:** Selected known secreted proteins with similar expression in sporozoites and tachyzoites.

		RPKM			FOLD CHANGE	
Gene ID	Description	SPZ	TZ	TZ+fzSPZ	SPZ/TZ	SPZ/TZ+fzSPZ
TGME49_297880	GRA2	197.3	229.4	208.3	0.9	0.9
TGME49_286450	GRA5	1327.5	1701.8	1814	0.8	0.7
TGME49_291890	MIC1	247	411.4	443.9	0.6	0.6
TGME49_255260	RON2	155.6	203	213.4	0.8	0.7
TGME49_229010	RON4	165.8	226.5	236.2	0.7	0.7
TGME49_262730	ROP16	146.7	187.2	169	0.8	0.9
TGME49_205250	ROP18	317.2	344	342.8	0.9	0.9
TGME49_308090	ROP5	547	526	473.8	1.0	1.2

We next examined the subsets of genes that show significant differences between the two developmental forms and observed that SRS44 and SRS35A are both much more abundantly expressed in the TZ sample (Table 6) whereas SRS52C is higher in SPZ (Table 5). SRS44 is also known as CST1 and is a key component of the tissue cyst wall [54]. SRS35A has not been directly studied but previously published microarray data indicate that it is very abundantly expressed in bradyzoites, moderately expressed in tachyzoites and barely if at all expressed in sporozoites [13]. SRS52C has not been further characterized.

Micronemes play a major role in invasion and of the genes encoding known or predicted microneme proteins, transcripts for AMA1, MIC2, M2AP, MIC10, and the newly characterized TgGAMA [55], were all significantly more abundant in sporozoites compared to tachyzoites (Table 8). On the other hand, there were 7 microneme proteins with increased expression in TZ relative to SPZ, including MIC12, MIC3, and MIC4.

**Table 8:** Differentially expressed genes encoding microneme proteins.

		RPKM				FOLD CHANGE
*Significantly higher expression in SPZ vs*. *TZ*						
Gene ID	Description	SPZ	TZ	TZ+fzSPZ	SPZ/TZ	SPZ/TZ+fzSPZ
**TGME49_201780**	**MIC2**	1511.1	664.3	638.8	**2.3**	**2.4**
**TGME49_250710**	**MIC10**	1281.3	593.2	695.8	**2.2**	**1.8**
**TGME49_214940**	**M2AP**	642.3	393.6	423	**1.6**	**1.5**
**TGME49_243930**	**TgGAMA**	160.7	101.1	102.2	**1.6**	**1.6**
**TGME49_255260**	**AMA1**	766.8	517.8	496.5	**1.5**	**1.5**
***Significantly higher expression in TZ vs*. *SPZ***						
**TGME49_267680**	**MIC12**	0	50	42	**inf**	**inf**
**TGME49_291890**	**MIC1**	247	411.4	443.9	**0.6**	**0.6**
**TGME49_200240**	**MIC17B**	30.9	52.9	56	**0.6**	**0.6**
**TGME49_208740**	**microneme protein, putative**	21.6	49.4	53.3	**0.4**	**0.4**
**TGME49_208030**	**MIC4**	141.6	348.8	326.5	**0.4**	**0.4**
**TGME49_319560**	**MIC3**	55.2	383.5	357.1	**0.1**	**0.2**
**TGME49_200250**	**MIC17A**	30.9	286.8	280	**0.1**	**0.1**

Ranked from lowest to highest fold-change in SPZ/TZ.

Only two annotated rhoptry proteins, namely ROP34 and ROP35, had higher expression in SPZ, showing respectively a 4- and 1.6-fold increase compared to the TZ samples (Table 9). These two proteins are members of the extensive ROPK kinase family, an extended set of proteins that include a mix of active and inactive protein kinases [56] and whose prototypic member is the predicted pseudokinase ROP2. The precise functions of ROP34 and ROP35 are not known but ROP35 was recently reported to be necessary for high cyst burdens during the chronic stage of a mouse infection [57].

**Table 9:** Differentially expressed genes encoding rhoptry proteins.

		RPKM			FOLD CHANGE	
*Significantly higher expression in SPZ vs*. *TZ*						
Gene ID	Description	SPZ	TZ	TZ+fzSPZ	SPZ/TZ	SPZ/TZ+fzSPZ
**TGME49_240090**	**ROP34**	936.6	216.9	241.5	**4.3**	**3.9**
**TGME49_304740**	**ROP35**	122.6	78.1	80.4	**1.6**	**1.5**
***Significantly higher expression in TZ vs*. *SPZ***						
**TGME49_311470**	**RON5**	130	190.8	192.4	**0.7**	**0.7**
**TGME49_315490**	**ROP10**	67.2	100.7	102.5	**0.7**	**0.7**
**TGME49_309590**	**ROP1**	1093.2	1670.4	1613.7	**0.7**	**0.7**
**TGME49_310010**	**RON1**	52.4	81.7	90.9	**0.6**	**0.6**
**TGME49_211290**	**ROP15**	170	279.5	271.8	**0.6**	**0.6**
**TGME49_315220**	**ROP14**	55.3	91.6	94.9	**0.6**	**0.6**
**TGME49_258580**	**ROP17**	172.1	325.1	310.7	**0.5**	**0.6**
**TGME49_266100**	**ROP41**	15	29.5	31.5	**0.5**	**0.5**
**TGME49_215775**	**ROP8**	606.6	1222.7	1263.5	**0.5**	**0.5**
**TGME49_308810**	**RON9**	37.1	80.1	67.1	**0.5**	**0.6**
**TGME49_297960**	**RON6**	46.4	101.1	91.8	**0.5**	**0.5**
**TGME49_215785**	**ROP2A**	735.1	1610.8	1562.3	**0.5**	**0.5**
**TGME49_294560**	**ROP37**	13.8	33	34.9	**0.4**	**0.4**
**TGME49_291960**	**ROP40**	88.5	213.5	225	**0.4**	**0.4**
**TGME49_223920**	**RON3**	74	196.3	175.2	**0.4**	**0.4**
**TGME49_258660**	**ROP6**	157.2	440.4	488.2	**0.4**	**0.3**
**TGME49_227810**	**ROP11**	33.9	95.4	102.7	**0.4**	**0.3**
**TGME49_252360**	**ROP24**	45.4	129.8	143	**0.3**	**0.3**
**TGME49_261750**	**RON10**	25.7	78.6	64.8	**0.3**	**0.4**
**TGME49_295110**	**ROP7**	441.2	1706.8	1658.9	**0.3**	**0.3**
**TGME49_203990**	**ROP12**	22.6	111.3	129.3	**0.2**	**0.2**
**TGME49_214080**	**toxofilin**	72.8	396.5	389.4	**0.2**	**0.2**
**TGME49_295125**	**ROP4**	152.3	913.2	902.1	**0.2**	**0.2**
**TGME49_242240**	**ROP19A**	3.3	61.8	78.2	**0.1**	**0.0**
**TGME49_262050**	**ROP39**	6.9	135.2	137.5	**0.1**	**0.1**
**TGME49_242110**	**ROP38**	3.6	116.6	116.1	**0.0**	**0.0**

Ranked from lowest to highest fold-change in SPZ/TZ.

Twenty-six known or predicted rhoptry genes had significantly lower expression in SPZ vs. TZ (Table 9). The precise function of most of these proteins is not known although many are also part of the ROPK family and are found at the parasitophorous vacuole membrane. One, ROP17, is known to play a role in neutralization of a potent anti-parasite defense mounted by immunity-related GTPases [58]. Another, ROP38, is known to be involved in down-regulation of host genes associated with MAPK signaling [59]. RON5 is included in this set but just met the criteria for significance and had a SPZ/TZ ratio of 0.7 which is similar to the values for its moving junction partners RON2 and RON4 which had SPZ/TZ ratios of 0.7 and 0.8, respectively but were included in Table 7 as not significantly different based on the statistical analysis.

In contrast to microneme and rhoptry genes, there were 25 genes out of the 48 annotated and/or reported to encode dense granule and dense granule-like proteins (ToxoDB, [33]) that had *higher* expressions in intracellular sporozoites compared to tachyzoites (Table 10). Interestingly, these include genes for the recently characterized GRA24, GRA16, GRA28, and GRA31 [33,60,61]. Notably, *GRA28* had the highest level of differential expression between the sporozoites and tachyzoites, with 12.5-fold higher expression in sporozoites than in tachyzoites (*q*-value = 0.77% at FDR 10%). *GRA15*, whose gene product is known to modulate NF-κB signaling in tachyzoites [62], has a significantly higher expression (2.2-fold, *q*-value = 0.77%) in infecting sporozoites compared to tachyzoites. Sporozoites also had higher levels of *MYR1*, which encodes a recently described protein necessary for translocation of dense granule proteins beyond the parasitophorous vacuole membrane (PVM) [63]. There were only 8 genes encoding dense granule proteins with higher expression in tachyzoites compared to infecting sporozoites (Table 10), with one isoform of *GRA11* [64] and *GRA36* [33] having the highest fold changes (~9- and 7-fold higher in the TZ, relative to SPZ samples, respectively). As yet, the functions of GRA11 and GRA36 are not known and so it is difficult to interpret these results in terms of the biology of the parasites. Furthermore, the gene for GRA39, recently shown to be critical for virulence of *Toxoplasma* type II PRU strain in mice [33], had a 1.9-fold increase in TZ relative to SPZ.

**Table 10:** Differentially expressed genes encoding dense granule or dense granule-like proteins.

		RPKM			FOLD CHANGE	
*Significantly higher expression in SPZ vs*. *TZ*						
Gene ID	Description	SPZ	TZ	TZ+fzSPZ	SPZ/TZ	SPZ/TZ+fzSPZ
**TGME49_231960**	**GRA28**	535.1	42.8	46.3	**12.5**	**11.6**
**TGME49_220240**	**GRA31**	490.5	124.6	121	**3.9**	**4.1**
**TGME49_208450**	**TgPI2**	875.8	223.6	284.9	**3.9**	**3.1**
**TGME49_230180**	**GRA24**	589.9	153.8	156.6	**3.8**	**3.8**
**TGME49_226380**	**GRA35**	433.4	132.9	139.4	**3.3**	**3.1**
**TGME49_208830**	**GRA16**	494.8	171	174.3	**2.9**	**2.8**
**TGME49_310780**	**GRA4**	1896.3	672.6	716.5	**2.8**	**2.6**
**TGME49_290700**	**GRA25**	398	157.9	143.9	**2.5**	**2.8**
**TGME49_254470**	**MYR1**	229.2	93.1	101.4	**2.5**	**2.3**
**TGME49_220950**	**MAF1b**	394.6	176.2	190.2	**2.2**	**2.1**
**TGME49_279100**	**MAF1a**	542	245.7	254.9	**2.2**	**2.1**
**TGME49_275860**	**GRA12 paralogue**	256.5	117.2	117.4	**2.2**	**2.2**
**TGME49_275470**	**GRA15**	106.4	49	58.4	**2.2**	**1.8**
**TGME49_240060**	**TgIST1**	433.8	202	205.7	**2.1**	**2.1**
**TGME49_254720**	**GRA8**	2376.3	1144.2	1167.2	**2.1**	**2.0**
**TGME49_312420**	**GRA38**	182.1	90.3	91.4	**2.0**	**2.0**
**TGME49_203310**	**GRA7**	1463.1	729.6	773.6	**2.0**	**1.9**
**TGME49_270250**	**GRA1**	10615.7	5360.4	5710.4	**2.0**	**1.9**
**TGME49_219810**	**GRA40**	113.8	62.1	61.2	**1.8**	**1.9**
**TGME49_239740**	**GRA14**	632.1	351.1	379.3	**1.8**	**1.7**
**TGME49_288650**	**GRA12**	1511.6	872.1	882.3	**1.7**	**1.7**
**TGME49_227280**	**GRA3**	1158.5	674.1	721.9	**1.7**	**1.6**
**TGME49_275440**	**GRA6**	1922.4	1166.8	1214.7	**1.6**	**1.6**
**TGME49_215220**	**GRA22**	446	273.6	265.2	**1.6**	**1.7**
**TGME49_247440**	**GRA33**	90.5	56.5	58.5	**1.6**	**1.5**
***Significantly higher expression in TZ vs*. *SPZ***						
**TGME49_222170**	**GRA17**	77.4	123.5	117.1	**0.6**	**0.7**
**TGME49_221210**	**cyclophilin**	307.6	496	560.9	**0.6**	**0.5**
**TGME49_289380**	**GRA39**	45.5	87.6	77.9	**0.5**	**0.6**
**TGME49_200010**	**GRA20**	27.2	79.1	80.3	**0.3**	**0.3**
**TGME49_203290**	**GRA34**	21.5	63.2	67.8	**0.3**	**0.3**
**TGME49_277270**	**NTPase II**	230.2	934.9	917.9	**0.2**	**0.3**
**TGME49_213067**	**GRA36**	9.2	65.3	70.5	**0.1**	**0.1**
**TGME49_237800**	**GRA11**	5.3	45.1	32.7	**0.1**	**0.2**

Ranked from lowest to highest fold-change in SPZ/TZ.

Notably, 331 of the 743 genes that have higher expression in sporozoites compared to tachyzoites during infection encode hypothetical proteins of completely unknown function and 85 of these hypothetical proteins are predicted to have a signal peptide (Table J in S1 File). Combined with the results with genes encoding known proteins, these findings are concordant with the similarities in host responses to sporozoites and tachyzoites reported here (i.e., higher expression of genes associated with Tumor Necrosis Factor alpha (TNFα) signaling via NF-κB), but suggest that these two developmental forms differ in the relative expression of various effectors that may have a role in subsequent invasion events.

### Genes involved in gene expression and cell division

Inner-membrane complex (IMC) proteins and functionally related proteins play a crucial role in parasite replication, motility, and host cell invasion. As might be expected, therefore, tachyzoites, which are the rapidly dividing form of *T*. *gondii* in the intermediate host, showed significantly higher RPKM levels for 27 out of the 34 IMC and IMC-associated genes [31,32] compared to the sporozoites (Table 11). In contrast, only transcripts for the IMC protein phosphatase, IMC2a, and the newly identified suture protein, ISC4 [32], had >2-fold higher expression in infecting sporozoites compared to tachyzoites.

**Table 11:** Differentially expressed genes encoding IMC proteins.

		RPKM			FOLD CHANGE	
*Significantly higher expression in SPZ vs*. *TZ*						
Gene ID	Description	SPZ	TZ	TZ+fzSPZ	SPZ/TZ	SPZ/TZ+fzSPZ
**TGME49_305930**	**ISC4**	40.6	17.4	17.2	**2.3**	**2.4**
**TGME49_228170**	**IMC2A**	294.2	126.5	133.6	**2.3**	**2.2**
***Significantly higher expression in TZ vs*. *SPZ***						
**TGME49_224530**	**IMC5**	0	39.6	38.6	**inf**	**inf**
**TGME49_271930**	**IMC20**	0	250.4	214.1	**inf**	**inf**
**TGME49_286580**	**IMC17**	0	41.7	39.8	**inf**	**inf**
**TGME49_217510**	**IMC19**	92.4	172.2	158.1	**0.5**	**0.6**
**TGME49_260820**	**ISP1**	55.1	120.3	131.3	**0.5**	**0.4**
**TGME49_253470**	**IMC13**	21.7	48.4	53.8	**0.4**	**0.4**
**TGME49_220930**	**ISC3**	16.3	56	61	**0.3**	**0.3**
**TGME49_219320**	**GAP50**	131.7	462.7	476.6	**0.3**	**0.3**
**TGME49_235380**	**AC5**	12.2	47	49.4	**0.3**	**0.2**
**TGME49_316540**	**ISP3**	12.4	49.5	63	**0.3**	**0.2**
**TGME49_250820**	**AC2**	9.7	45.9	32.5	**0.2**	**0.3**
**TGME49_249850**	**GAP40**	77.5	378.6	372.9	**0.2**	**0.2**
**TGME49_223940**	**GAP45**	24.1	198.1	174.6	**0.1**	**0.1**
**TGME49_295360**	**IMC18**	12.5	103.9	105.7	**0.1**	**0.1**
**TGME49_219170**	**ISC2**	4.5	38.9	32.5	**0.1**	**0.1**
**TGME49_232030**	**IMC21**	14.7	144.7	140.1	**0.1**	**0.1**
**TGME49_308860**	**AC3**	9.3	95.4	104.4	**0.1**	**0.1**
**TGME49_214880**	**AC4**	5	52.2	52.8	**0.1**	**0.1**
**TGME49_258470**	**IMC24**	66.9	713.9	731	**0.1**	**0.1**
**TGME49_230210**	**IMC10**	28.3	316.9	307.8	**0.1**	**0.1**
**TGME49_226220**	**IMC9**	2.5	32.7	35.2	**0.1**	**0.1**
**TGME49_220270**	**IMC6**	8.6	138.2	124.1	**0.1**	**0.1**
**TGME49_216000**	**IMC3**	20	392.8	356.2	**0.1**	**0.1**
**TGME49_235340**	**ISC1**	2.4	58.4	56.1	**0.0**	**0.0**
**TGME49_316340**	**IMC22**	8.1	293.7	257.3	**0.0**	**0.0**
**TGME49_231640**	**IMC1**	14.8	581.3	549.5	**0.0**	**0.0**
**TGME49_225690**	**AC7**	0	41.3	40	**0.0**	**0.0**

Ranked from lowest to highest fold-change in SPZ/TZ. Inf = infinity.

There are 68 annotated AP2 domain-containing transcription factors in the *T*. *gondii* genome. Some of these are cell-cycle-regulated [65] and have been implicated in the transcriptional regulation of interconversion of tachyzoites to bradyzoites [66,67] and of virulence determinants, including *ROP18* [68]. Six genes encoding AP2-domain transcription factors had higher expression in sporozoites relative to tachyzoites, including TgAP2X-2 with an ~4-fold increase, whereas 17 such genes were significantly lower in the sporozoites (Table 12). The latter set included TgAP2IX-9, which prevents stage conversion of tachyzoite to bradyzoite [66], as well as the cell-cycle-regulated proteins TgAP2XII-9 and TgAP2XI-1 [65].

**Table 12:** Differentially expressed genes encoding AP2 transcription factors.

		RPKM			FOLD CHANGE	
*Significantly higher expression in SPZ vs*. *TZ*						
Gene ID	Description	SPZ	TZ	TZ+fzSPZ	SPZ/TZ	SPZ/TZ+fzSPZ
**TGME49_225110**	**AP2X-2**	**62.3**	**15.8**	**13.2**	**3.9**	**4.7**
**TGME49_227900**	**AP2X-1**	**42**	**17.4**	**19.4**	**2.4**	**2.2**
**TGME49_247700**	**AP2XII-4**	**88.8**	**45.6**	**42.7**	**1.9**	**2.1**
**TGME49_203050**	**AP2VIIa-6**	**26**	**13.7**	**14.6**	**1.9**	**1.8**
**TGME49_272710**	**AP2VIII-4**	**37.7**	**24.2**	**23.1**	**1.6**	**1.6**
**TGME49_310900**	**AP2XI-2**	**106.7**	**72.7**	**68.5**	**1.5**	**1.6**
***Significantly higher expression in TZ vs*. *SPZ***						
**TGME49_208020**	**AP2Ib-1**	0	16.7	15	**inf**	**inf**
**TGME49_217700**	**AP2XII-2**	0	19.4	16.8	**inf**	**inf**
**TGME49_309410**	**AP2XI-1**	0	22	25.9	**inf**	**inf**
**TGME49_203710**	**AP2VIIa-4**	15.9	47	41.3	**0.3**	**0.4**
**TGME49_288950**	**AP2IX-4**	7.3	21.8	21.7	**0.3**	**0.3**
**TGME49_315760**	**AP2XI-4**	5.3	17.8	15.1	**0.3**	**0.4**
**TGME49_264485**	**AP2IX-3**	2.1	7.2	6	**0.3**	**0.4**
**TGME49_253380**	**AP2III-2**	14.5	56.8	52.5	**0.3**	**0.3**
**TGME49_215570**	**AP2X-11**	4.5	21.1	24.5	**0.2**	**0.2**
**TGME49_282210**	**AP2VIIa-8**	7.8	38.3	33.4	**0.2**	**0.2**
**TGME49_240900**	**AP2VI-2**	2.8	16.6	13.2	**0.2**	**0.2**
**TGME49_237425**	**AP2X-6**	0.7	6.7	6.5	**0.1**	**0.1**
**TGME49_271200**	**AP2VIII-5**	2.7	28.1	32.6	**0.1**	**0.1**
**TGME49_306620**	**AP2IX-9**	0.9	15	14.5	**0.1**	**0.1**
**TGME49_289710**	**AP2IX-5**	2.7	64.5	64.1	**0.0**	**0.0**
**TGME49_318470**	**AP2IV-4**	1	28	28.5	**0.0**	**0.0**
**TGME49_251740**	**AP2XII-9**	0	42.4	45.6	**0.0**	**0.0**

Ranked from lowest to highest fold-change in SPZ/TZ. inf = infinity.

### Genes encoding metabolic enzymes

The metabolic state of intracellular sporozoites has not previously been investigated and so we analyzed our transcriptomic data for clues to how these parasites might compare to tachyzoites in this regard. Using the KEGG metabolic pathway enrichment module available on ToxoDB, we observed that the 999 genes whose expression was significantly lower in sporozoites compared to tachyzoites were enriched for genes associated with glycolysis and gluconeogenesis, fatty acid biosynthesis, as well as several other metabolic pathways (Bonferroni adjusted *p*-value <0.05, Table 13). Consistent with the apicoplast’s function in *de novo* fatty acid synthesis using the FASII pathway [69], several genes associated with this organelle were increased in TZ compared to SPZ, including ACP [70,71], which had RPKMs of about 117 and 0 in TZ vs. SPZ, respectively (Table I in S1 File). Additionally, transcripts for the apicoplast resident proteins PDH-E2 and ACC1 [72–74] had significantly higher expression in TZ with ~5- and 2.5-fold increase relative to SPZ, respectively (Table I in S1 File). On the other hand, the genes with higher expression in sporozoites than in tachyzoites show some degree of significant enrichment in riboflavin, nicotinate and nicotinamide, purine, and pyrimidine metabolisms (Table 13). In all cases except glycolysis/gluconeogenesis, however, the Bonferroni-adjusted *p*-values were between 0.001 and 0.05 making conclusions about possible implications for the metabolic state of these respective stages tentative until further examined by more direct means.

**Table 13:** Metabolic pathways differentially enriched in intracellular sporozoites vs. tachyzoites.

*Significantly enriched in SPZ vs*. *TZ*					
KEGG Pathway name (# genes in reference)	# Genes	Fold enrichment	Odds ratio	P-value	Bonferroni
Purine metabolism (287)	35	1.95	2.41	7.99x10^-5^	1.44x10^-3^
Pyrimidine metabolism (197)	27	2.19	2.59	1.19x10^-4^	2.15x10^-3^
Nicotinate and nicotinamide metabolism (146)	22	2.41	2.77	1.74x10^-4^	3.13x10^-3^
Riboflavin metabolism (88)	14	2.54	2.77	1.94x10^-3^	3.48x10^-2^
***Significantly enriched in TZ vs*. *SPZ***					
Glycolysis / Gluconeogenesis (39)	20	5.51	6.16	1.92x10^-8^	5.56x10^-7^
Citrate cycle (TCA cycle) (27)	11	4.38	4.63	1.91x10^-4^	5.55x10^-3^
Limonene and pinene degradation (43)	13	3.25	3.45	5.6x10^-4^	1.62x10^-2^
Fatty acid biosynthesis (44)	13	3.18	3.37	6.72x10^-4^	1.95x10^-2^
Pyruvate metabolism (34)	11	3.48	3.66	9.51x10^-4^	2.76x10^-2^
Lysine degradation (73)	17	2.50	2.68	1.06x10^-3^	3.07x10^-2^

From KEGG metabolic pathway enrichment on ToxoDB

## Discussion

Of the three developmental forms capable of infection, *Toxoplasma* sporozoites have been the least studied. In this work, we used RNASeq and an *in vitro* model of infection of the intestine to determine the host response to sporozoite infection as well as the state of the parasite’s own transcriptome. Comparing these data to those from infection with tachyzoites and previous studies on day 10 oocysts allowed us to identify host and parasite genes that may be specifically involved in these different stages of a *Toxoplasma* infection.

Our findings indicate that the primary response of rat IECs to *Toxoplasma gondii*, whether as sporozoite or tachyzoite, involves an NF-kB-like inflammatory response. This response is marked by a significant increase in expression of genes encoding proteins associated with NF-kB signaling, including chemokines and inflammatory cytokines. For instance, *Ccl20*, *Cxcl1*, and *Ccl2*, which were among the highly expressed genes during *Toxoplasma* infection, are involved in recruitment and activation of immune cells, such as neutrophils and lymphocytes [75,76]. The absence of alterations in the host transcriptome in the presence of frozen-thawed sporozoites indicates that inactive sporozoites do not provide pathogen-associated molecular patterns (PAMPs), at least not ones recognized by these IECs; instead, it appears that active parasite processes lead to the observed host gene activation. The slightly stronger host response measured in the TZ vs. SPZ cultures could be due to either an inherent property of tachyzoites or the slightly higher MOI for the tachyzoite infections (0.26 vs. 0.18 for the sporozoites); we cannot discriminate between these possibilities at present.

Whether sporozoites down-regulate particular host pathways could not be determined from our analysis because the low MOI in our experiments means 70–80% of the IECs in any given experiment were uninfected and so, even if a given gene is completely off in an infected cell, the transcript abundance would be reduced by only 20–30% in the population which is less than the experimental error in these RNASeq-based assays. Achieving a higher MOI may be difficult with the available amounts of infectious sporozoites but the advent of single-cell transcriptomic analyses will help circumvent this problem [77].

To identify sporozoite-specific effectors that may contribute to the transcriptomic changes observed in infected IECs, we also profiled the transcriptomes of the infecting sporozoites and tachyzoites. Consistent with the NF-κB results, *GRA15* and *GRA25*, which encode two dense granule proteins known to modulate NF-κB activity and chemokine secretion (Ccl2 and Cxcl1), respectively, during infection with tachyzoites [62,78], were expressed in both forms albeit at somewhat higher levels in the infecting sporozoites. Transcripts encoding other well-characterized tachyzoite effectors [51,53], namely ROP16, ROP5, and ROP18 were also all expressed at similar levels in both sporozoites and tachyzoites. GRA24, GRA16, and the newly identified GRA28, have all been shown to localize to the host nucleus [33,79] and all three had a significantly higher expression in infecting sporozoites compared to tachyzoites. Whether these effector proteins localize and function in a similar fashion during infection with sporozoites remains to be studied.

Of perhaps greater importance is that our results show 85 genes encoding putatively secreted, uncharacterized proteins that have higher expression in the sporozoites compared to tachyzoites. It is likely like that some of these proteins are effectors that contribute to the ability of sporozoites to initiate an infection in the intestine of a new host. The need for differential expression of these genes could reflect the different cell type being infected by a sporozoite, almost exclusively an intestinal endothelial cell, vs. a tachyzoite, any of a large number of cells in many organs ranging from neurons in the CNS to myocytes in muscle. Alternatively, these differences in effector repertoire could result from the fact that the role of sporozoites is to initiate an infection in a presumptively naïve host whereas tachyzoites disseminate the infection up to and beyond the time of a potent anti-tachyzoite immune response. The effectors needed for these different stages and locations of an infection could be very different.

Analysis of the infecting sporozoite transcriptome revealed a significant increase in the expression of several AP2 transcription factors, suggesting that these play a role in the regulation of the differences in gene expression seen between sporozoites and tachyzoites. Further support for this inference comes from the recent findings that AP2 transcription factors, such as TgAP2XI-4, which was found here to be increased in tachyzoites, are involved in the transcriptional regulation of tachyzoite-to-bradyzoite interconversion [67]. Interestingly, TgAP2X-1 and TgAP2XII-4, which we saw to be increased in sporozoites, were recently shown to contribute to type I tachyzoites’ growth *in vitro* [80], suggesting an important role in both these developmental stages.

The different transcript levels for a large number of genes from extracellular D10 sporulated oocysts vs. the intracellular sporozoites from 5-month old oocysts used in this study were surprising. It could be that after sporulation is complete, there are major changes in the transcriptome if the sporozoites do not quickly invade a new host. This possibility is supported by the observation that transcript levels for sporoSAG, sporoAMA1, and sporoRON2 are already much lower in D10 compared to D4 sporulated oocysts based on RNAseq (ToxoDB) and published microarray data [13] available on ToxoDB (Table 4). Alternatively, the differences observed could be due to the fact that we are looking 8 hours post-infection. As previously reported, invaded sporozoites transition to the tachyzoite form about 12 hours after infection [34] and so our finding few if any transcripts for several genes that were high in D10 oocysts could indicate that these transcripts are rapidly degraded as part of the differentiation process. What fraction of the differences reflects this vs. the further development of the oocysts prior to their use in these experiments, as mentioned above, is not currently known.

Overall, our results show that, at least within the constraints of the *in vitro* model used here, and although the response of IECs to sporozoite infection is qualitatively similar to that seen with tachyzoites, many transcriptomic differences are seen, especially on the parasite side. Future studies will seek to characterize these genes and determine the role they play in enabling sporozoites to initiate a successful infection.

## Supporting information

S1 File Supplementary tables described below. **Table A: Summary of average total RNASeq reads mapped. Table B: Numbers of raw reads mapped to rat genes or exons from CLC Genomics. Table C: Numbers of raw reads mapped to *Toxoplasma* genes or exons from CLC Genomics. Table D: Final list of 16416 rat genes with average exon reads included in analysis pipeline with RPKM values and fold changes.** Only genes with ≥ 5 exon reads are included. **Table E: Final list of 6469 *Toxoplasma* genes with average adjusted exon reads that were included in analysis pipeline with RPKM values and fold-changes.** Only genes with ≥ 5 exon reads are included. **Table F: Results of SAMseq statistical analyses for all pairwise comparisons performed. Table G: Rat genes with increased expression during infection with tachyzoites.** Ranked by ratio of read numbers for TZ/Mock. **Table H: *Toxoplasma* genes with higher expression in intracellular sporozoites compared to tachyzoites.** Ranked by ratio of read numbers for SPZ/TZ; inf = infinity; Only genes with ≥ 5 exon reads are included. **Table I: *Toxoplasma* genes with higher expression in intracellular tachyzoites compared to sporozoites.** Only genes with at least 20 reads in TZ are included in the table. Ranked by ratio of read numbers for TZ/SPZ; inf = infinity. **Table J: Significantly higher genes encoding hypothetical proteins with predicted signal peptides in SPZ vs. TZ.** Ranked by ratio of read numbers for SPZ/TZ; inf = infinity.
(XLSX)
